# Effects of striatal transplantation of cells transfected with GDNF gene without pre- and pro-regions in mouse model of Parkinson’s disease

**DOI:** 10.1186/s12868-016-0271-x

**Published:** 2016-06-10

**Authors:** A. Revishchin, L. Moiseenko, N. Kust, N. Bazhenova, P. Teslia, D. Panteleev, V. Kovalzon, G. Pavlova

**Affiliations:** Laboratory of Neurogenetic and Developmental Genetic, Institute of Gene Biology, Russian Academy of Sciences, Vavilova Str., 34/5, Moscow, Russia 119334; Ltd Apto-pharm, Kolomensky Road, 13A, Moscow, Russia 115446; Department of Higher Nervous Activity, Faculty of Biology, M.V. Lomonosov Moscow State University, Lenin Hills d. 1, pp. 12, Moscow, Russia 119234; A.N. Severtsov Institute of Ecology and Evolution, Russian Academy of Sciences, 33 Leninskij Prosp., Moscow, Russia 119071; A.N. Severtsov Institute of Ecology and Evolution, Russian Academy of Sciences, Moscow, Russia; Research Institute of General Pathology and Pathophysiology, Russian Academy of Sciences, Moscow, Russia 125315

**Keywords:** Neurotrophic factor, GDNF, Parkinson’s disease, Sleep-wakefulness cycle, Substantia nigra

## Abstract

**Background:**

Previously, we have shown that transgenic cells bearing the GDNF gene with deleted pre- and pro-regions (mGDNF) can release transgenic GDNF. The medium conditioned by transgenic cells with mGDNF induced axonal growth in rat embryonic spinal ganglion in vitro. Here we demonstrate a neurotrophic effect of mGDNF on PC12 cells in vitro as well as its neuroprotective effect on dopaminergic neurons in the substantia nigra pars compacta in vivo as indicated by improved motor coordination and sleep-wakefulness cycle in the MPTP mouse model of Parkinson’s disease.

**Results:**

HEK293 cells were transfected with a vector encoding an isoform of the human GDNF gene with deleted pre- and pro-regions (mGDNF). This factor in the medium conditioned by the transfected cells was shown to induce axonal growth in PC12 cells. The early Parkinson’s disease model was established by injection of the dopaminergic pro-neurotoxin 1-methyl-4-phenyl-1,2,3,6-tetrahydropyridine (MPTP) into C57Bl/6 mice. Transgenic HEK293/mGDNF/GFP cells were transplanted into the striatum (caudate-putamen) of experimental mice. The sleep-wakefulness cycle was studied by continuous EEG and motor activity monitoring 1 and 2 weeks after MPTP injection. After the experiment, the motor coordination of experimental animals was evaluated in the rotarod test, and dopaminergic neurons in the substantia nigra pars compacta were counted in cross-sections of the midbrain. MPTP administration lowered the number of tyrosine hydroxylase immunopositive cells in the substantia nigra pars compacta, decreased motor coordination, and increased the total wake time during the dark period. The transplantation of HEK293/mGDNF cells into the caudate-putamen 3 days prior to MPTP injection smoothed these effects, while the control transplantation of HEK293 cells showed no notable impact.

**Conclusions:**

Transplantation of transgenic cells with the GDNF gene lacking the pre- and pro-sequences can protect dopaminergic neurons in the mouse midbrain from the subsequent administration of the pro-neurotoxin MPTP, which is confirmed by polysomnographic, behavioral and histochemical data. Hence it is released from transfected cells and preserves the differentiation activity and neuroprotective properties.

## Background

Glial cell line-derived neurotrophic factor (GDNF) promotes the survival and differentiation of neurons and glial cells [1–3]. This GDNF activity can be useful in the treatment of neuronal degeneration and loss of differentiation typical for a number of neurodegenerative diseases. GDNF has a pronounced neuroprotective effect on dopaminergic neurons and spinal motoneurons [4] and induces axonal growth [5].

GDNF is expressed in both neurons and astrocytes [6, 7]. It was proposed that elevated GDNF synthesis in astrocytes promotes neuronal survival after ischemic [8] and excitotoxic damage [6]. The importance of GDNF for the maintenance of neuronal viability is also confirmed by the transplantability of cerebral tissues in GDNF-deficient mice. Dopaminergic neurons of GDNF^−/−^ mouse embryos transplanted into the dorsal striatum of wild type mice cannot survive and innervate the striatum after MPTP-induced degeneration of their endogenous dopaminergic neurons [9]. The significance of GDNF as a neurotrophic factor was also confirmed by a sharp reduction of dopaminergic sprouting in the injured striatum after antisense inhibition of GDNF expression [10]. Protective effect of GDNF on dopaminergic neurons was demonstrated in several models of Parkinson’s disease [11–15].

The human GDNF gene contains six exons and generates five isoforms [16]. The encoded GDNF mRNAs include the full-length pre-(α)pro-GDNF transcript and the pre-(β)pro-GDNF, the latter is shorter by 78 bp in region of the pro-domain. The protein encoded by pre-(α)pro-GDNF is released from the cell via the conventional pathway through the Golgi apparatus [17]. At the same time, the release of the shorter protein encoded by pre-(β)pro-GDNF is largely mediated by secretogranin II and Rab3A-positive vesicles and, thus, bypasses the Golgi apparatus. Kust et al. [1] demonstrated that the deletion of the pre- and pro-regions of the GDNF gene does not affect the transgenic factor release from transfected cells. Moreover, the deletion of both pre- and pro-regions enhances the trophic activity of GDNF. Spinal ganglia cultured in the presence of medium conditioned by cells transfected with mGDNF demonstrated active growth of β-3-tubulin-positive axons by day 4 of culture [1].

Here, we studied the effect of transgenic mGDNF encoded by the GDNF gene with deleted pre- and pro-regions in PC12 cells in vitro. Then, the effect of mGDNF-producer cells on the survival of dopaminergic neurons in the mouse substantia nigra was evaluated in vivo using the conventional 1-methyl-4-phenyl-1,2,3,6-tetrahydropyridine (MPTP) model of Parkinson’s disease [18]. This model was used repeatedly for the study of neuroprotective substances, neurotoxin being administrated in many cases after the neuroprotectors [16, 19]. The MPTP effect depends on the dose and administration mode. Here, we used a single subcutaneous administration of 40 mg/kg MPTP, which induces an early clinical stage of Parkinson’s disease [19].

The effect of mGDNF-producing transgenic cells was evaluated using the rotarod test for motor coordination of experimental mice [20]. In addition, we implemented a test evaluating early abnormalities of brain function through the changes in the sleep-wakefulness cycle. Parkinson’s disease is accompanied by a wide range of sleep-wakefulness cycle abnormalities observed in 45–92 % patients. In particular, many patients demonstrate prolonged nighttime awakenings and reduced NREM and REM sleep [21, 22]. In the MPTP model of early Parkinson’s disease, experimental mice also demonstrate increased activity and reduced NREM and REM sleep at nighttime [23], i.e., in the same period of pineal melatonin production when the corresponding sleep disorders are observed in patients [24]. So, this model is adequate for studying the effects of various biochemical factors in early Parkinson’s disease.

In this work it was demonstrated that transgenic factor mGDNF lacking pre- and pro-sequences is not only secreted by cells and stimulates neurite growth in vitro but also demonstrates neuroprotective properties in the neurotoxic model of Parkinson’s disease which had been shown several times for the full length GDNF molecule. We have found that mGDNF is more secreted by transfected cells than the pre-pro-GDNF. We confirm by our work that transgenic factor mGDNF stimulates neurite growth and neural differentiation of PC12 cells in vitro. Using the experiments with the injection of transgenic cells to the mice striatum and subsequent system administration of MPTP, we have found that the GDNF isoform retains its neurotrophic properties also in vivo when the factor is secreted into active intracerebral medium which is quite different from the cultural one. Modified transgenic factor secreted by the cells injected into striatum makes indirect retrograde effect on substantia nigra cells. This indicates that mGDNF can be used for treating nerve tissue degeneration observed in a number of nervous system disorders.

## Methods

### Genetic constructs and primers

The mGDNF construct with deleted pre- and pro-regions and with an EGFP tag was generated by introducing a HindIII site, a Kozak sequence, and an extra start codon upstream of the “m” part as well as by removing the stop codon and introducing a BamHI site in the 3′ region of mGDNF using PCR. The following primers were employed: *Gdnf*^*Hind*III^(F) 5′-AAGCTTCCACCATGTCACCAGATAAACAA-3′ and *Gdnf*^*Bam*H1^(R)5′-GGATCCCAG ATACATCCACACCTTTTAGCGG-3′. The plasmid pGEM-T Easy (Promega) containing the full-length human GDNF cDNA [25] was used as the template. PCR was performed using the Tersus polymerase (Evrogene) and the following program: 94 °C for 1.5 min; 25 cycles of 94 °C for 15 s, 57 °C for 20 s, and 72 °C for 15 s; and final 72 °C for 10 min. The resulting 354 bp (118 amino acids) fragment was isolated from agarose gel using a Qiaquick Gel Extraction Kit (Qiagen) and cloned into pGEM-T Easy (Promega). The HindIII/BamHI fragment of the resulting construct pGEM/m*Gdnf* was cloned into the corresponding sites of pEGFP-N1 (Clontech). For the control we used construct with pre-pro-GDNF, which were prepared using the primers T3 (F) 5′-ATTAACCCTCACTAAAGGGA-3′ и Gdnf ^*Bam*H1^ 5′-TGGATCCCAGA TACACCACACCTTTTAGCGG-3′. This construct was obtained according to the protocol described elsewhere [1].

### Transgenic cell cultures

Human Embryonic Kidney 293 (HEK293) cell line was obtained from the Russian Cell Culture Collection (Institute of Cytology of the Russian Academy of Sciences, St. Petersburg, Russia). HEK293 cells were cultured in complete DMEM (PanEko) supplemented with 10 % fetal calf serum (Perbio HyClone), 2 mM l-glutamine (PanEko), and 10 µg/ml gentamicin (PanEko) at 37 °C with 5 % CO_2_ in 25 cm^2^ Costar flasks. At 70–80 % confluence, the cells were transfected with the generated constructs using ExGen 500 (Fermentas). The transfected clones were selected with 0.4 mg/ml geneticin (G418, Sigma) for 10 days, after which G418-resistant clones were analyzed by PCR for the inserted gene sequences. The transgene expression was verified by RT-PCR with the corresponding primers.

### RT-PCR

Total RNA was isolated using Tri reagent (Sigma), treated with DNAseI (Thermo Scientific) (1 U per 1 μg RNA), and used for reverse transcription with M-MuLV Reverse Transcriptase and oligo (dT) primer. The efficiency of reverse transcription was evaluated by PCR with the primers for GAPDH (F, 5′-GGCCATGAGGTCCACCACCCTGTTGCTGTA-3′; R, 5′-CCCCTGGCCAAGGTCATCCATGACAACTT-3′) and for neomycin (F, 5′-ATGATTGAACAAGATGGATT-3′; R, 5′-TCAGAAGAACTCGTCAAGAA-3′. RNA not subjected to reverse transcription was used as a negative control. The efficiency of transgene expression was evaluated by PCR with the following primers: *Gdnf*^*Hind*III^(F) 5′-AAGCTTCCACCATGTCACCAGATAAACAA-3′ and *gfp* (R) 5′-AATAAAGCTTGCATGGCGGTAATACG-3′. The PCR amplification program consisted of 94 °C for 2 min; 30 cycles of 93 °C for 10 s, 58 °C for 20 s, and 72 °C for 30 s; and final 72 °C for 5 min.

### ELISA

The 24-h culture media of transgenic HEK293/mGDNF/GFP, transgenic HEK293/pre-pro-GDNF/GFP, and HEK293 (control) were used in the assay. GDNF was quantified using the GDNF Emax ImmunoAssay System (Promega) and a microplate reader Synergy 4 (Tecan) according to the manufacturer’s protocol.

### Analysis of mGDNF effect on PC12 cells

PC12 cells are a clonal cell line derived from a pheochromocytoma of the rat adrenal medulla. They are used as a model for the study of neuronal differentiation [26]. PC12 (ATCC CRL1721) cells were tested for neuronal sprouting after the exposure to conditioned medium containing GDNF with deleted pre- and pro-regions. Transgenic HEK293 cells were plated on 25 cm^2^ flasks and, after reaching confluence of about 60 %, the complete medium was replaced with serum-free DMEM. After 72 h of culture at 37 °C, the conditioned medium was harvested and filtered through a 0.22 nm filter.

PC12 cells were plated at 3 × 10^4^ cells/well on four-well plates coated with rat tail type I collagen in RPMI1640 containing 10 % horse serum, 2 mM l-glutamic acid, and 100 µg/ml streptomycin. After 4 h of culture, the medium was replaced with that conditioned by transgenic HEK/mGDNF/GFP cells. The medium conditioned by untransfected HEK293 cells for 72 h was used as control. The concentration of chimeric GDNF proteins was evaluated in the media conditioned by transgenic HEK293 cells for further analysis. This concentration was confirmed by ELISA. Based on the obtained data, the concentration of ~1.25 ng/ml was used to analyze the chimeric protein activity in vitro. The following controls were used: (1) medium conditioned by HEK293 cells transgenic for GFP; (2) medium supplemented with 1.25 ng/ml recombinant GDNF (SantaCruz); (3) unconditioned complete culture medium. After a 3-day culture in conditioned or control medium, PC12 cells were fixed in 4 % formaldehyde and analyzed by phase contrast microscopy under an inverted microscope Olympus IX81. Then these cells were stained using the primary polyclonal antibodies against β-3-tubulin (Abcam) and secondary Cy2-conjugated donkey anti-rabbit antibodies. After washing in PBS, cells were mounted in glycerol and analyzed under an inverted fluorescent microscope Olympus IX81. The proportion of cells with axons equal to or longer than the small diameter of the cell was counted on phase contrast and fluorescent images using the ImageTool software (UTHSCSA) [27]. Five counts including 100–120 cells were carried out for each studied construct. The obtained data were analyzed using the SPSS software (IBM, USA).

### Cell transplantation and electrode implantation for electroencephalographic analysis of the sleep-waking cycle

The neuroprotective effect of transgenic mGDNF encoded by the GDNF gene with deleted pre- and pro-regions on the viability of dopaminergic neurons in the substantia nigra pars compacta was studied in the early Parkinson’s disease model. Transgenic cells were injected into the striatum (the caudate nucleus/putamen region) of mice 3 days prior to subcutaneous administration of 40 mg/kg of the proneurotoxin MPTP.

Four groups of animals were studied:

1. Animals transplanted with transgenic HEK293/mGDNF/GFP cells 3 days prior to MPTP injection (N = 10).
2. Animals transplanted with HEK293/GFP cells without the GDNF gene 3 days prior to MPTP injection (N = 10).
3. Animals transplanted with transgenic HEK293/mGDNF/GFP cells with no subsequent MPTP injection (N = 5).
4. Animals injected with MPTP without preliminary cell transplantation (N = 11).

All in vivo experiments were approved by the Ethics Committee of Moscow State University. Animals anesthetized by chloral hydrate were placed in a stereotaxic frame. Transgenic HEK293/mGDNF/GFP cells were injected into the striatum of C57BL/6j mice at the age of 2.5–3 months weighing 25–30 g (groups 1 and 3). A suspension containing about 150,000 cells in 1 µl of Hanks solution was bilaterally injected into the brain. The injection was performed slowly (over a period of 3 min) with a microsyringe at coordinates AP 0 mm and ML 2.5 mm (the caudate nucleus/putamen region). The needle was inserted to a depth of 2.5 mm and withdrawn in steps to a depth of 1.5 mm. HEK293/GFP cells were injected similarly into animals of group 3. Next, four epidural electrodes were permanently implanted for electroencephalographic (EEG) monitoring in the frontal and parietal neocortex. The reference electrode was placed on the nasal bone. Animals of group 4 were not transplanted with cells, while the electrodes were implanted as described above. After implantation, animals were placed into small individual soundproof boxes equipped with highly sensitive module video cameras attached to a video recorder, which was consequently connected to a PC via USB port. Animals were kept under a 12/12 light/dark cycle (09–21 h, bright white light; 21–09, dim red light), temperature 22–24 °C, and free access to food and water.

Each animal was attached via a flexible cable to a miniature digital two-channel biopotential amplifier supplied with a three-axis accelerometer (for mechanographic monitoring) attached via a flexible spring to an independent power supply, which was consequently attached to rotatable hook in the box ceiling. This construction allowed three-axis motions of the amplifier plate (30 × 28 × 7 mm in size and 8 g in weight) in response even to faint movements of the animal. The digitization frequencies of the EEG and accelerometer signals were 250 and 50 Hz, respectively. The signal from wireless amplifiers was transmitted via Bluetooth channel to the recording computer and visualized using the modified open-source software *EDF browser* [28]. The EEG and accelerometer bandwidths were set equal to 1–20 and 1–12 Hz, respectively. The animal behavior and motor activity were also monitored by video tracking. Animals of group 4 had a 7-day recovery period after implantation. After this period, the EEG (background) and mechanographic monitoring was continuously performed for 24 h. Such monitoring was repeated 7 and 14 days after MPTP administration. Experimental conditions allowed no long recovery period and, thus, no background EEG recording for animals of the first three groups. Accordingly, only the dynamics of the sleep-wakefulness cycle was evaluated 7 and 14 days after MPTP administration in comparison to the baseline records in group 4 animals.

### MPTP administration and analysis of its effects

Three days after cell transplantation and electrode implantation, animals of groups 1, 2, and 4 were subcutaneously injected 40 mg/kg of the dopaminergic proneurotoxin MPTP (Sigma, St. Louis, MO, USA). One and two weeks later, EEG and mechanographic (by accelerometer) records were made. The polysomnograms (EEG + mechanogram) obtained for all animals were visually evaluated for 20-s epochs. Wake as well as NREM and REM sleep stages were identified using the standard criteria: wake, desynchronized cortical EEG, 5–7 Hz hippocampal theta-rhythm in the parietal (hippocampal projection) EEG, and high accelerometer signal; NREM sleep, high delta and sigma EEG activity and low accelerometer signal; REM sleep, very high and regular 6–8 Hz theta-rhythm in the parietal-hippocampal EEG and zero accelerometer signal [29]. The data obtained were analyzed by nonparametric statistical methods using the GraphPad/Prism-4.02 software (Friedman and Kruskal–Wallis analysis of variance, post hoc Dunn’s test, and Wilcoxon and Mann–Whitney tests).

After the experiment, the motor coordination of experimental animals was tested on a Rotarod (TSE Systems, Bad Homburg, Germany). Animals were exposed to 6 rpm for 10 min, after which the rotational speed was increased in steps of 1 rpm every 30 s until the animal fell onto the tray with wood shavings. The time of falling and velocity were recorded.

Fifteen days since MPTP administration (18 days after transgenic cells injection) the animals were anesthetized again and perfused through the heart with PBS and then with 4 % formaldehyde in PBS. The brain was isolated, fixed again in formaldehyde for 12 h at 4 °C, and soaked in 30 % sucrose in PBS for 24 h. The cryotome coronal sections of the brain (40 μm) were mounted in PBS. Four series of sections were prepared for each brain. The sections in antifreeze solution were stored at −20 °C until staining. Every fourth section containing the substantia nigra was immunohistochemically stained for tyrosine hydroxylase (TH) using monoclonal antibodies (Sigma) diluted 1:200 in PBS with 2 % normal horse serum, 0.5 % Triton X-100, and 0.01 % sodium azide (Sigma). Free-floating sections were incubated in primary antibodies at 4 °C for 48 h. After incubation in biotinylated horse anti-mouse antibodies diluted 1:100 (Vector Labs, Burlingame, CA, USA) and then in ABC diluted 1:200 (Vector Labs), the standard staining for peroxidase was performed using PBS with 0.03 % diaminobenzidine (Sigma) and 0.01 % hydrogen peroxide. The stained sections were mounted on slides in 50 % glycerol and covered with slips. TH-immunopositive (TH+) cells were quantified on an Olympus IX81 microscope with a computer-controlled motorized stage (Märzhäuser, Wetzlar, Germany) and an *Olympus DP72* digital camera (Olympus, Münster, Germany). Cells were counted using the Cell* software (Olympus Soft Imaging Solution, Münster, Germany). After obtaining an overview of the compact part of the substantia nigra (SNC) and the ventral tegmental area (VTA) at a low magnification (10× objective), TH+ cells were counted using the optical fractionator method [30] at a higher magnification (40× objective). The 50 × 50 µm counting frame was shifted in 200 µm steps in both X- and Y-directions within the ventral part of the midbrain. At each position of the counting frame, the focal plane was shifted in the Z direction by 30 µm. An uninformed operator counted unstained nuclei of TH+ cells in counting frames.

### Western blot hybridization

To know how long the transgenic HEK293/mGDNF/GFP cells can survive in striatum and produce fusion protein mGDNF/GFP we used Western Blot analysis. Two, three, five and eighteen days since administration of transgenic cells we cut out fragments of mouse striatum in a volume of 3–4 mm^3^ which include the site of injection, then powdered them in a liquid nitrogen and lysed in the following buffer (100 µl per 106 cells): 60 mM Tris–HCl (pH 6.8), 25 % (v/v) glycerol, 2 % SDS, 5 % (v/v) 2-mercaptoethanol, and 0.01 % (w/v) bromophenol blue. Protein concentration was determined by Bradford assay and 40 µg protein samples were loaded onto a 10 % gel and analyzed by SDS-PAGE. Proteins were transferred to a Hybond ECL membrane (Amersham, Buckinghamshire, UK) using a Mini trans-Blot cell (Bio-Rad #170-3930) according to the manufacturer’s instructions in the buffer containing 25 mM Tris, 192 mM glycine, and 20 % (v/v) methanol, pH 8.3 at 100 V for 1 h. The membrane was stained with Ponceau Red and thoroughly rinsed with TBS-T buffer. Then the membrane was incubated on a shaker in 5 % defatted milk powder in TBS-T at room temperature for 30 min and washed three times with TBS-T for 5 min. GDNF was detected using monoclonal antibodies against GDNF (D20, Santa Cruz Biotechnology, Dallas, USA). The membrane was incubated with the primary antibodies at 4 °C overnight and washed with TBS-T. Incubation with the secondary peroxidase-conjugated antibodies (1:3000) was carried out at room temperature for 1 h, and the membrane was washed with TBS-T. GDNF detection was performed using an ECL Advance Western Blotting Detection Kit (Amersham) according to the manufacturers’ instructions. In each group there were 3 mice with the same surviving time since the cell administration. The intensity of the bands were measured using ImageJ [31].

### Statistical analysis

Data are presented as mean ± SEM. The statistical analysis was performed using the SPSS software. The values were compared by one-way ANOVA followed by Tukey’s multiple comparisons test. Statistical significance was accepted at p < 0.05.

## Results

### Quantitative analysis of GDNF released from transgenic cells

**Fig. 1:**
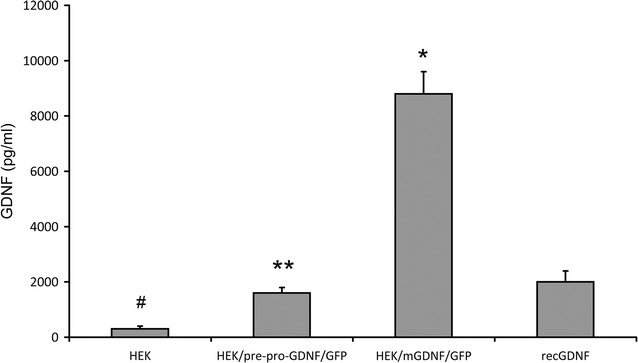
Quantitative analysis of GDNF in medium conditioned by transgenic and control untransfected HEK293 using ELISA. (HEK) medium conditioned by untransfected HEK293 cells; (pre-pro-GDNF/GFP) medium conditioned by HEK293 cells transfected with pre-pro-GDNF/GFP; (mGDNF) medium conditioned by HEK293 cells transfected with mGDNF/GFP; (recGDNF) medium fortified with recombinant GDNF (SantaCruz) diluted to a concentration of 2 ng/ml. Data are presented in pg/ml as mean ± SEM (n = 3). Differences between values are significant at *p < 0.01 as compared with all other values. **p < 0.05 as compared with HEK293. ^#^p < 0.05 as compared with recGDNF (one-way ANOVA)

The transgenic culture of human embryonic kidney cells HEK293 producing mGDNF/GFP was obtained using the protocol described elsewhere [1]. The release of the fusion mGDNF/GFP protein from transgenic HEK293 cells was evaluated by ELISA. Each experiment was done in triplicates. The medium conditioned by untransfected HEK293 cells was used as control. HEK293 cells were cultured in DMEM containing 10 % fetal serum, 2 mM l-glutamine at 37 °C in a CO_2_ incubator. GDNF was quantified using the GDNF Emax ImmunoAssay System. mGDNF/GFP was shown to be released to the culture medium of the transgenic cells. The level of released mGDNF/GFP was much higher than that of the full-length pre-pro-GDNF/GFP (Fig. 1). Likewise, mGDNF level in the conditioned medium was also much higher compared to pre-pro-GDNF/GFP. The observed experimental differences were significant at p < 0.05 (one-way ANOVA).

### The influence of conditioned media containing mGDNF/GFP on neurite outgrowth in PC12 cells

**Fig. 2:**
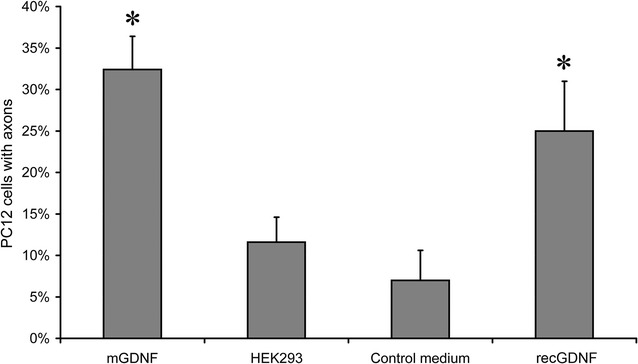
Percentage of PC12 cells with axons after 3-day culture in media conditioned by transgenic cells or in control media. (recGDNF) medium fortified with 1.25 ng/ml pre-pro-GDNF (SantaCruz); (HEK293/mGDNF) medium conditioned by HEK293/mGDNF/GFP with mGDNF/GFP adjusted to 1.25 ng/ml; (HEK293) medium conditioned by untransformed HEK293 cells; (control medium) unconditioned medium—serum-free DMEM. *p < 0.05 as compared with HEK293 and control medium

The efficiency of conditioned media containing mGDNF/GFP was analyzed using PC12 cells. After 3-day culture in conditioned medium with GDNF lacking the pre- and pro-regions as well as with recombinant GDNF, the proportion of cells with axons (exceeding the neuronal body size) was significantly higher than that in control cultures at p < 0.05 (Fig. 2). One-way ANOVA indicated significant differences between the control cells cultured in medium conditioned by untransformed HEK293 cells and those cultured in the normal unconditioned medium. The proportion of cells with axons cultured in medium conditioned by transgenic HEK293/mGDNF/GFP cells was substantially and significantly higher than that in control cells cultured in unconditioned medium. The difference between cells cultured in media conditioned by transgenic HEK293/mGDNF/GFP and HEK293/pre-pro-mGDNF/GFP was also significant. The highest proportion of cells with axons was observed in cells cultured in medium conditioned by HEK293/mGDNF/GFP.

**Fig. 3:**
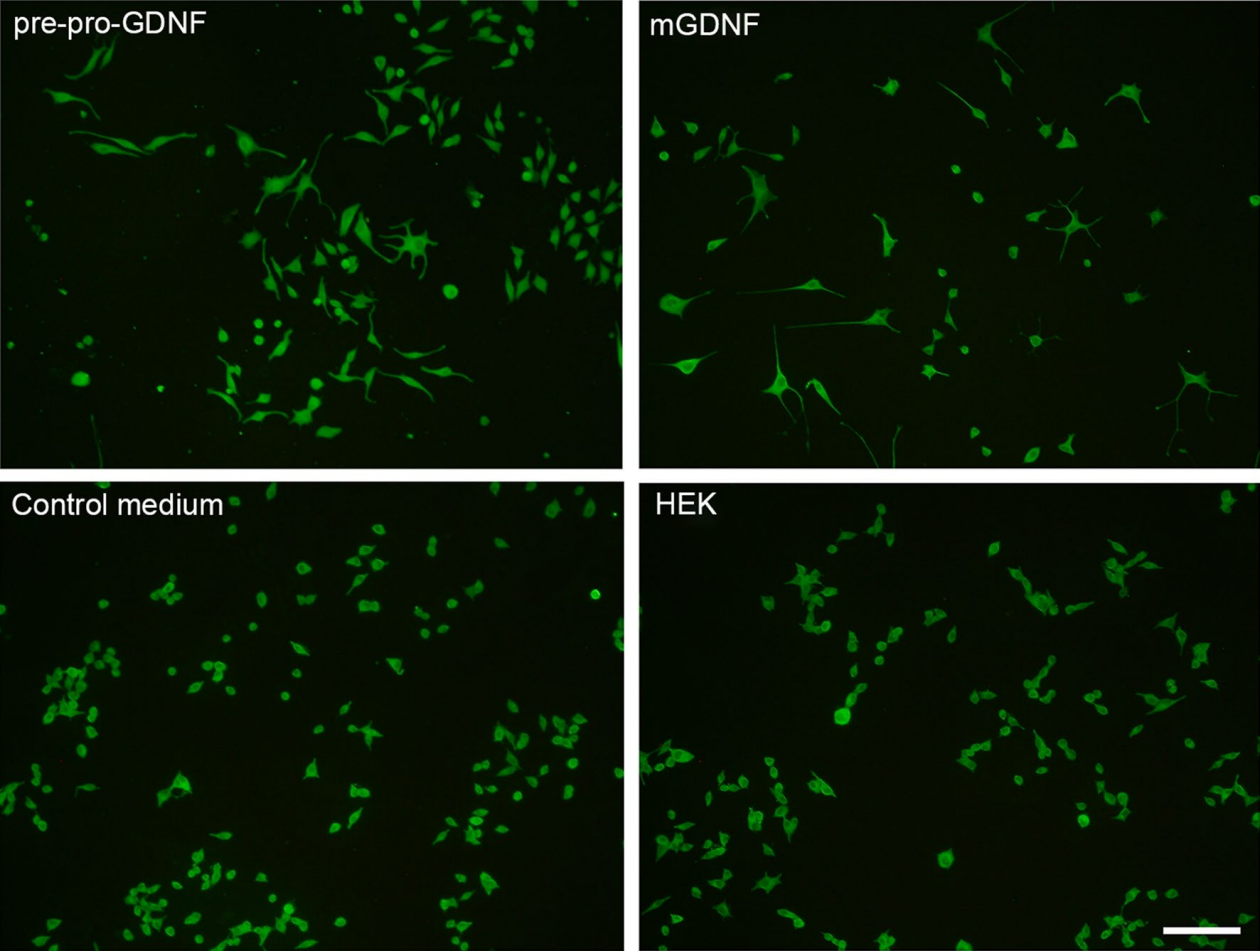
PC12 cells immunocytochemically stained for β-tubulin after culture in media conditioned by transgenic cells or in control media. (pre-pro-GDNF) media conditioned by HEK293 cells transfected with pre-pro-GDNF/GFP; (mGDNF) media conditioned by HEK293 cells transfected with mGDNF/GFP and mGDNF/GFP; (control medium) unconditioned medium—serum-free DMEM; (HEK) media conditioned by untransfected HEK293. Cells cultured in media conditioned by transfected cultures demonstrate a significantly higher proportion of cells with axons. *Scale* 100 µm (*right lower corner*)

Figure 3 demonstrates immunohistochemical staining of PC12 cells exposed to media conditioned by HEK293 cells transfected with pre-pro-GDNF/GFP and mGDNF/GFP, by untransfected HEK293 (HEK), and in unconditioned medium. Thus, the removal of the pre- and pro-regions did not affect the transport of this GDNF modification from the cell and improved the inductive properties of the factor.

## Analysis of protective properties of the GDNF modification in vivo using the mouse MPTP model of Parkinson’s disease

### Histological studies of changes in the substantia nigra and ventral tegmental area

**Fig. 4:**
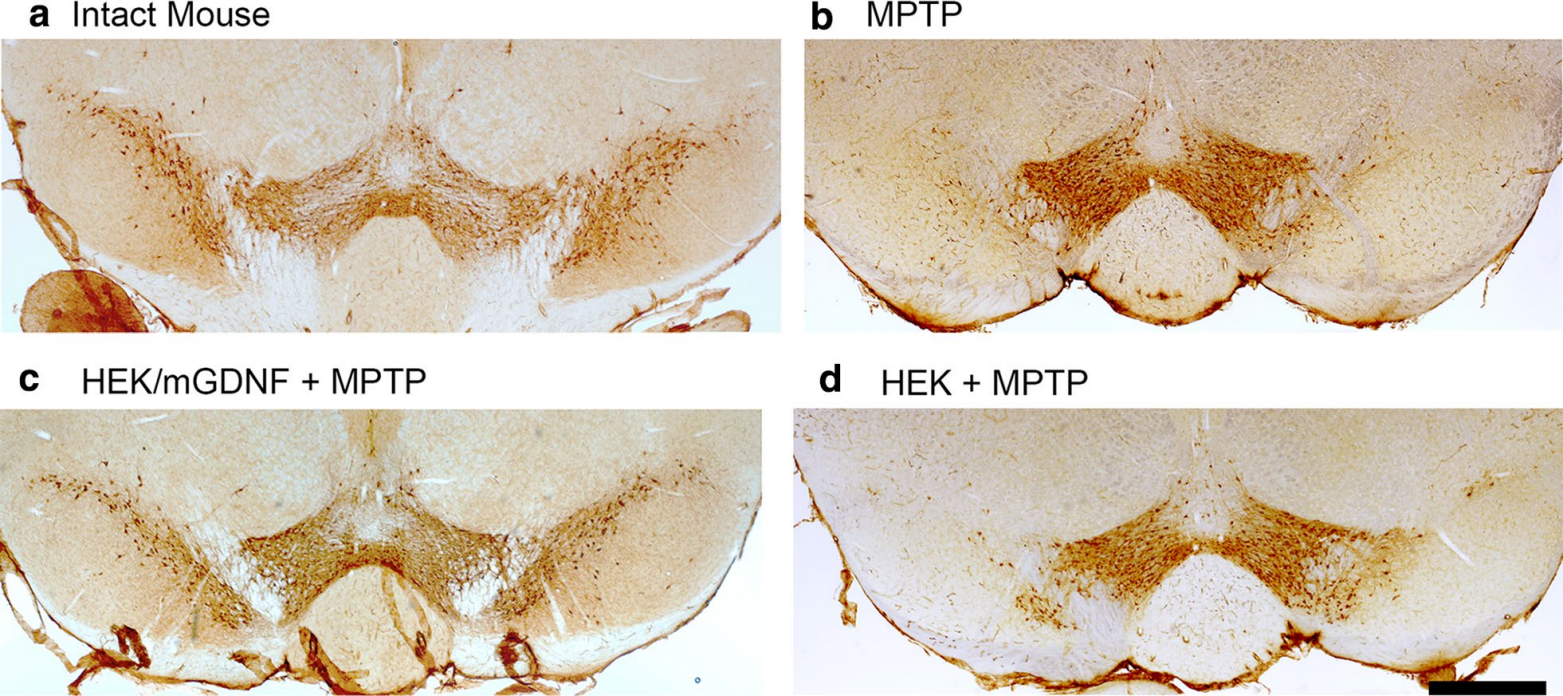
Representative micrographs of coronal sections of the ventral midbrain in an intact and MPTP treated animals. **a** Intact mouse, **b** a mouse after MPTP injection; **c** a mouse transplanted with HEK293/mGDNF/GFP cells 3 days prior to MPTP injection; **d** a mouse transplanted with HEK293/GFP cells 3 days prior to MPTP injection. *Scale* 500 µm (*right lower corner*)

**Fig. 5:**
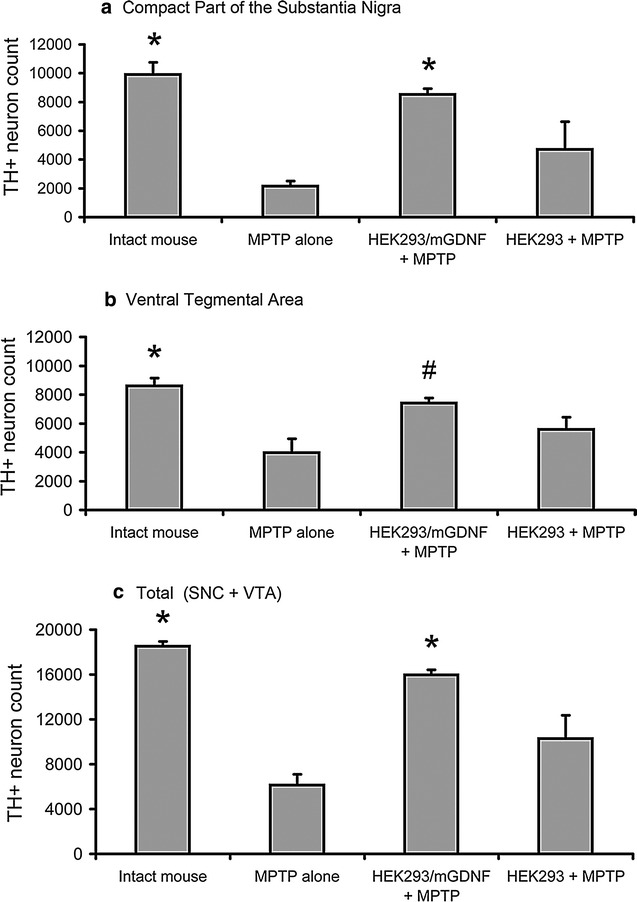
TH+ neuron counts in the ventral midbrain (SNC and VTA) in intact and MPTP treated animals. **a** TH+ neuron counts in the substantia nigra pars compacta. **b** TH+ neuron counts in the ventral tegmental area. **c** Total number of TH+ neuron in ventral midbrain. (MPTP) group 4 animals injected with MPTP only; (HEK293/mGDNF + MPTP) group 1 animals transplanted with HEK293/mGDNF/GFP 3 days prior to MPTP injection; (HEK293 + MPTP) group 2 animals transplanted with HEK293/GFP cells prior to MPTP injection. *p < 0.05 as compared with MPTP only and HEK293/GFP. ^#^p < 0.05 as compared with MPTP only

Analysis of sections prepared from animals sacrificed 17 days after the injection of 40 mg/kg of MPTP demonstrated significant changes in the number of dopaminergic neurons in the substantia nigra pars compacta between the experimental and control animals (Figs. 4, 5). Control animals injected with MPTP alone demonstrated a significant decrease in the number of TH+ neurons in the ventral midbrain. In SNC, the number of TH+ neurons decreased by 78 %; in VTA, by 54 % relative to control; while the overall number of TH+ neurons decreased by 67 % (Fig. 5). The number of TH+ neurons in these midbrain structures in animals transplanted with cells expressing the GDNF gene with deleted pre- and pro-sequences (HEK293/mGDNF/GFP + MPTP) was significantly higher than that in animals of two control groups administered MPTP alone or after transplantation with cells without transgenic GDNF (HEK293/GFP + MPTP).

**Fig. 6:**
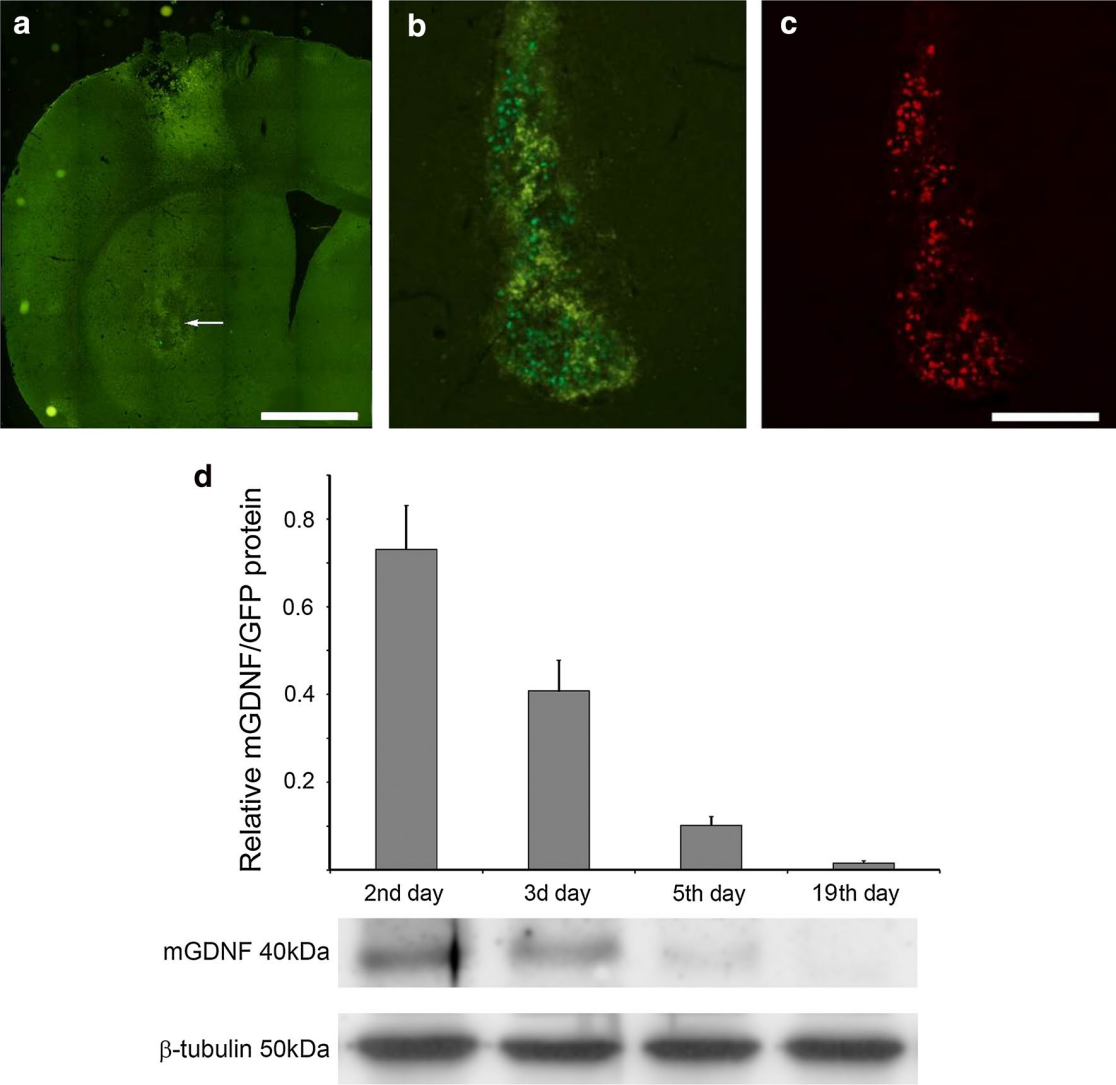
The time-dependent mGDNF level in the striatum after HEK/mGDNF/GFP transplantation. **a**–**c** Representative fluorescence micrographs of coronal sections of the striatum in the transplantation site. **a** GFP-expressing cells were almost missing in the injection site (indicated by the *arrow*) in the caudate-putamen of animals sacrificed 19 days after transplantation of transgenic cells containing the modified GDNF. One can see two GFP-expressing cells. **b** mGDNF/GFP-producing cells 3 days after transplantation into the striatum of mouse brain. **c** The same section immunohistochemically stained for GDNF (visualized by Texas red-conjugated secondary antibodies). *Scale* in **a** 1 mm, in **b**, **c** (shown on **c**) 200 μm. **d** The results of Western blot analysis of mGDNF/GFP expression in striatum 2, 3, 5, 18 days after cell administration

The caudate-putamen locus where HEK293/mGDNF/GFP and HEK293/GFP cells were injected was examined on the brain sections crossing the striatum using fluorescence microscopy. In all cases, the transplantation loci were in the middle part of the caudate-putamen (Fig. 6a). At the same time, nearly no GFP-positive cells were found in the stratum of experimental animals, which can be due to a long period of time passed after transplantation. Control animals transplanted with HEK293/mGDNF/GFP or HEK293/GFP cells were sacrificed 3 days after transplantation. These controls demonstrated GFP-positive transgenic cells in the transplantation site (Fig. 6b). Immunohistochemical analysis using antibodies against GDNF demonstrated that HEK293/mGDNF/GFP cells expressed transgenic GDNF 3 days after transplantation into the stratum (Fig. 6c).

### Analysis of motor coordination

**Fig. 7:**
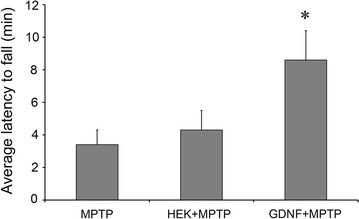
Rotarod test of mice of groups 1, 2, and 4. The results for mice of group 4 injected with MPTP only (MPTP, N = 11), group 2 transplanted with HEK293/GFP cells prior to MPTP injection (HEK + MPTP, N = 10), and group 1 transplanted with HEK293/mGDNF/GFP cells prior to MPTP injection (mGDNF + MPTP, N = 10). Ordinate, rotarod speed when animals fell down after a 30 s exposure normalized to group 4 (100 %), mean ± SEM. *Difference from group 4 significant at p < 0.05

In our experiments, mice of all groups could retain on the rotarod at 6 rpm for 10 min. As the speed increased, experimental groups demonstrated substantial differences in the threshold speed of the animal falling down. Mice of group 1 transplanted with cells expressing modified GDNF without the pre- and pro-regions prior to MPTP administration demonstrated the best results remaining on the rotarod at 21 rpm, while mice of groups 2 and 4 transplanted with cells without the GDNF gene or not transplanted fell down at the speed of 12–14 rpm (Fig. 7). The results for group 1 significantly differed from those for groups 4 and 2 (p < 0.05).

### EEG and behavioral analysis

**Fig. 8:**
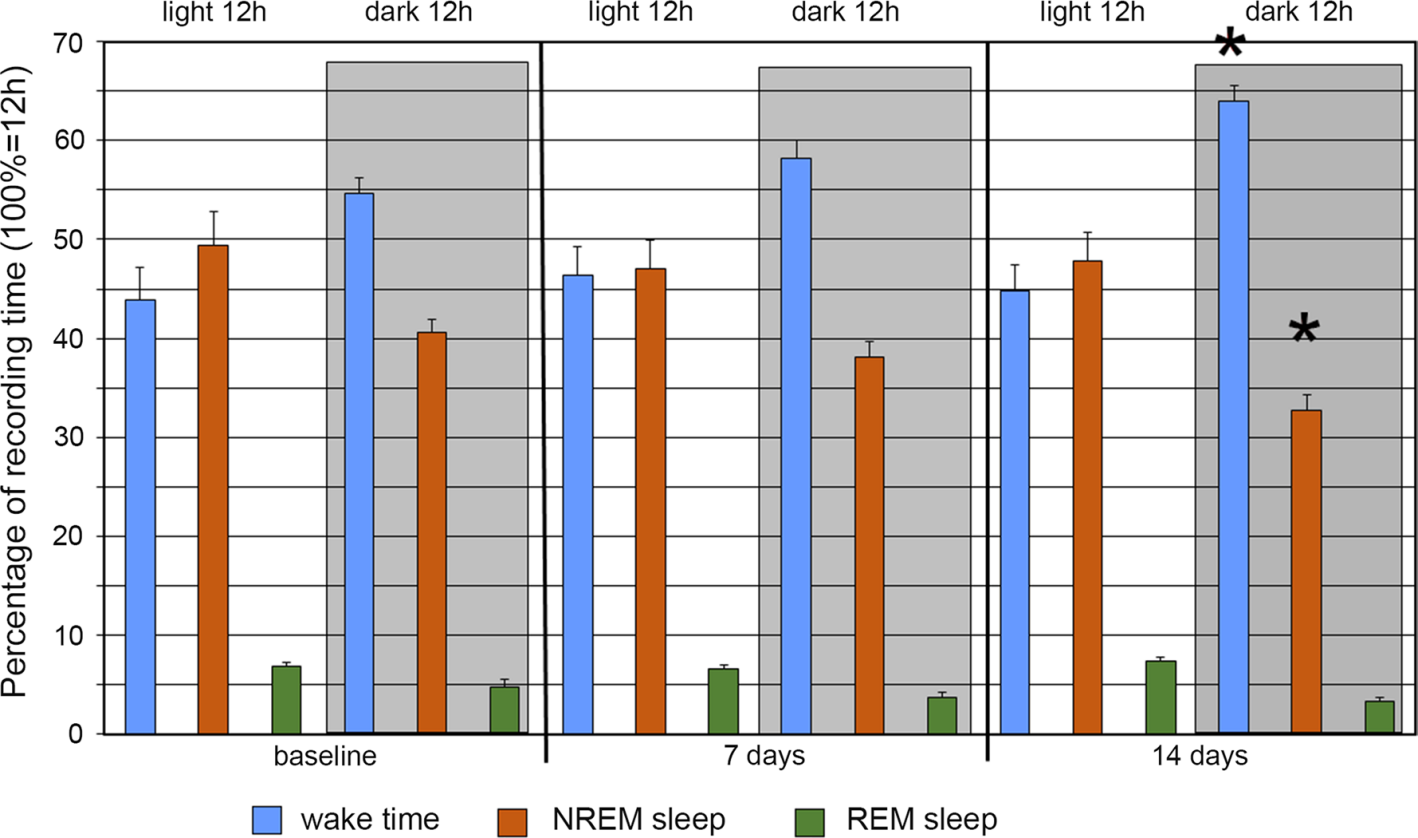
Changes in the sleep-wakefulness cycle in group 4 mice after administration of proneurotoxin MPTP. Percentage (12 h = 100 %) of wake time (*blue bars*), NREM sleep time (*brown bars*), and REM sleep time (*green bars*) during the light and dark (*shaded rectangle*) 12-h periods before (*baseline*) as well as 7 and 14 days after MPTP administration. *Changes from baseline significant at p < 0.01 (N = 9)

EEG was recorded 7 and 14 days after MPTP administration. The injection of this proneurotoxin into control mice (group 4) gradually increased the wake time and decreased the NREM sleep time during the dark period. These changes observed on day 7 became significant by the day 14 (Figs. 4, 5a). The REM sleep time did not significantly change and demonstrated only a trend to decrease. No notable changes were observed during the light period (Fig. 8).

**Fig. 9:**
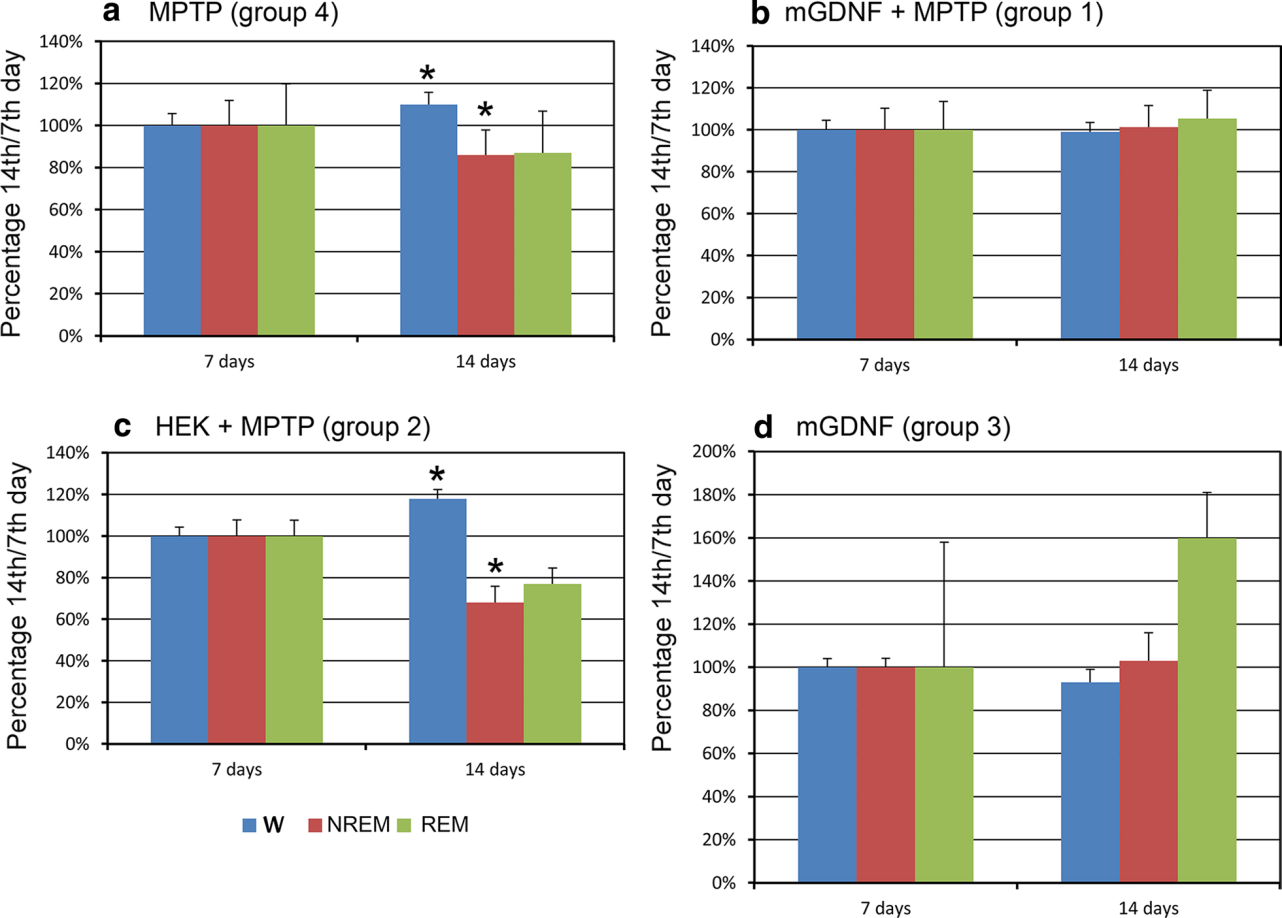
Changes in sleep–wakefulness cycle at the 14th day since administration as compared to the 7th day. Proportions (normalized to day 7 after MPTP administration, 100 %) of wake time (*blue bars*), NREM sleep time (*brown bars*), and REM sleep time (*green bars*) during the dark 12-h period 7 and 14 days after MPTP administration. **a** Increased wake time and decreased NREM sleep time after MPTP administration in group 4 (N = 9); **b** no changes in the sleep–wakefulness cycle in group 1 mice transplanted with HEK293/mGDNF/GFP cells prior to MPTP administration (N = 9); **c** changes in the sleep-wake cycle in group 2 mice transplanted with HEK293/GFP cells prior to MPTP administration; **d** no changes in the sleep-wakefulness cycle in three mice transplanted with HEK293/mGDNF/GFP cells (N = 3), high REM sleep data dispersion is due to the low number of animals in the group. *Changes are significant at p < 0.05

Transplantation of transgenic HEK293/mGDNF/GFP cells into animals of group 1 prior to MPTP injection dampened these effects (Fig. 9b). If cells without the GDNF gene were transplanted (group 2), no dampening was observed, and the pattern of changes was similar to that of group 4 (Fig. 9c). Animals of group 3 demonstrated no significant differences from the baseline of group 4 (Fig. 9d).

## Discussion

The capacity of GDNF to induce axonal growth in neuronal precursors in vivo suggests that it can be used to inhibit neurodegenerative process and prevent neuronal death following ischemic stroke or during neurodegenerative diseases [2–5, 7, 8, 26]. However, clinical trials in Parkinson’s disease patients after intracerebroventricular administration of recombinant GDNF demonstrated minor or no clinical improvements. A significant effect was initially observed after a direct infusion of recombinant GDNF into the striatum [32]; however, it has not been confirmed by a phase II double-blind trial conducted by Amagen so further clinical trials were discontinued. Nevertheless, well-documented protective properties of GDNF tempt both scientific and pharma specialists to find a way to using it as a neurodegenerative drug. For instance, MedGenesis Therapeutix and Pfizer made an agreement for joint development of methods for GDNF application and the convection enhanced delivery method in Parkinson’s disease.

One of possible approaches is a change of recombinant GDNF molecule through a modification of the vector bearing its gene. The presence of 2 splice variants of the matrix RNA of GDNF gene can indicate their different functions [33–36]. In our pervious study [1] we have studied the cell secretion and functional activity of various GDNF isoforms. For this purpose, we transfected HEK293 cells using plasmid constructions involving 4 different GDNF gene isoforms: a modification with pre- and pro-sequences (pre-pro-Gdnf); modification with pre- sequence only (pre-Gdnf); modification with pro- sequence only (pro-Gdnf); modification without both pre- and pro-sequences (mGdnf). In vitro experiments demonstrated that deleting pro-sequences as well as simultaneous deleting both pre- and pro-sequences of GDNF do not prevent the factor’s secretion by the cell and do not decrease its neurotrophic activity.

Here, we used ELISA to demonstrate a substantial and significant improvement in the release of transgenic GDNF without pre- and pro-regions from transfected cells compared to that with intact pre- and pro-regions. In vitro experiments on PC12 cells demonstrated that the differentiation activity of transgenic GDNF with deleted pre- and pro-regions is as high as that of recombinant GDNF with intact pre- and pro-regions. So GDNF can be secreted from the cell even at the absence of the sequences which are necessary for regulation of the process of its secretion. Uncontrolled intensive secretion of GDNF may be useful for the construction of gene-cellular therapeutic drugs if the high concentration of the gene product must be achieved at the site of transgenic cell transplantation. The data obtained suggest that the genetic constructs containing GDNF with deleted pre- and pro-regions can become more efficient in gene therapy compared to full-length GDNF variants.

Important advantage of gene-deleted constructions without pre- and pro-sequences is inability to secreting GDNF pro-forms from the transgenic cells. Mature forms of many neurotrophic factors (NGF, BDNF, NT3) realize their neuroprotective and differential activity via tyrosine kinase receptors (TrkA, TrkB and TrkC). At the same time their pro-forms which may be also synthesized and secreted from neurons and glia induce apoptosis via p75NTR-sortilin signaling cascades [37–41]. Regarding the GDNF, it is known that in a case of overexpression after plasmid transfection unprocessed proGDNF can be also secreted from the cell [17, 42]. proGDNF activity in the brain is insufficiently studied, however it is known that its expression increased in the ventral part of midbrain in MPTP mouse model of Parkinson’s disease. In rat LPS model, proGDNF is expressed in nigral neurons and glia [43]. One may propose that proGDNF may be involved into pathogenesis of Parkinson’s disease and does not counteract pathological disorders. In this case the lack of the pro-sequence in a transgene will be in favor of the therapeutic effect of transfected cells.

The main purpose of the study is to check up neuroprotective properties of mGDNF construction in vivo when the mature protein is secreted not to the cultural but the active intercellular medium. We used the mouse MPTP model of Parkinson’s disease to evaluate neuroprotective properties of the construct with mGDNF in vivo. MPTP injection considerably decreased the number of TH+ neurons in the ventral midbrain of experimental animals. The transplantation of transgenic cells with the GDNF gene lacking the pre- and pro-regions into the caudate-putamen of mice 3 days prior to MPTP injection substantially neutralized the negative impact of the proneurotoxin. Under these conditions, the MPTP induced a smaller decrease in the number of TH+ neurons in experimental animals compared to those transplanted with no cells or cells expressing no transgenic GDNF. It should be noted, after all, that the transplantation of HEK293 cells containing no transgenic GDNF prior to MPTP administration also has some neuroprotective effect on dopaminergic neurons in the substantia nigra pars compacta and the ventral tegmental area. This is indicated by a significant increase in calculated TH+ neurons as compared to animals not subjected to transplantation. A similar protective effect of untransfected cells was described by Cunningham and Su [12]. Following the authors, one can putatively attribute this effect to a weak trophic impact of injected cells or to the effect of factors released by brain cells injured by injection of a large volume of cells.

The test on rotating rod (rotarod) is conventionally used to evaluate functional disturbances in this model [20]. This test demonstrated a smoothing of MPTP effect on the motor abilities of experimental animals after the transplantation of cells transfected with the GDNF gene without the pre- and pro-sequences. A minor attenuation of MPTP effect was also observed after the transplantation of cells without GDNF into the striatum; however, this effect did not significantly differ from control with MPTP injection without cell transplantation.

We also evaluated the effect of cells releasing GDNF without the pre- and pro-regions on the non-motor consequences of MPTP administration described previously [23]. In present experiments as well as the cited publication, mice demonstrated increased motor activity, elongated total wake time, and shortened NREM and REM sleep time during the darkness (period of diurnal activity) within two weeks after systemic MPTP administration. These changes are observed in the same circadian period of pineal melatonin production when the corresponding sleep disorders are observed in Parkinson’s disease patients [24]. The transplantation of cells with transgenic GDNF lacking the pre- and pro-regions into the striatum 3 days prior to MPTP administration efficiently antagonized these changes. In contrast to the MPTP control, the sleep-wake ratio during the dark period was the same on days 7 and 14. At the same time, the transplantation of control HEK293/GFP cells into the same striatum region had no effect on the MPTP-induced changes in the sleep-wakefulness cycle (group 2). The transplantation of transgenic mGDNF-expressing cells without subsequent MPTP administration also had no effect on the sleep-wakefulness cycle (group 3). It may be concluded that namely the GDNF protein released by HEK293/mGDNF/GFP cells is the factor antagonizing the neurotoxic impact of MPTP in our experiments (group 1).

Non-motor manifestations of Parkinson’s disease attract increasing attention since it is becoming more and more clear that their understanding can contribute to early diagnosis of this illness [44]. Such manifestations of Parkinson’s disease include sleep disorders nighttime insomnia, daytime sleepiness, and REM behavior disorders, which are early predictors of Parkinson’s disease that can appear years or even decades before motor disturbances [22]. The data obtained in this work demonstrate that analysis of the changes in sleep-waking cycle is an adequate approach to verify early stages of Parkinson’s disease in model animals as well as to test anti-Parkinson’s disease effects of various biochemical factors.

## Conclusions

Thus, transplantation of transgenic cells with the GDNF gene lacking the pre- and pro-sequences can protect dopaminergic neurons in the mouse midbrain from the influence of subsequent administration of the proneurotoxin MPTP, which is confirmed by polysomnographic, behavioral (rotarod) and histochemical data. This work confirms our previous data that the product of the construct bearing the GDNF gene without the pre- and pro-sequences is not only released from transfected cells but also preserves the neurotrophic, differentiation, and neuroprotective properties.

## Abbreviations

GDNF: glial cell line-derived neurotrophic factor
mGDNF: mature GDNF
MPTP: 1-methyl-4-phenyl-1,2,3,6-tetrahydropyridine
GFP: green fluorescent protein
TH: tyrosine hydroxylase
TH+: tyrosine hydroxylase immunopositive
ELISA: enzyme-linked immunosorbent assay
REM sleep: rapid eye movement sleep
NREM sleep: non-rapid eye movement sleep
EEG: electroencephalography
SNC: substantia nigra pars compacta
VTA: ventral tegmental area
